# Retraction Note: Predicting the compressive strength of polymer-infused bricks: A machine learning approach with SHAP interpretability

**DOI:** 10.1038/s41598-026-56869-9

**Published:** 2026-06-09

**Authors:** Sathvik Sharath Chandra, Rakesh Kumar, Archudha Arjunasamy, Sakshi Galagali, Adithya Tantri, Sujay Raghavendra Naganna

**Affiliations:** 1 Department of Civil Engineering, Dayananda Sagar College of Engineering, Bengaluru, 560111 India; 2 Department of Artificial Intelligence and Machine Learning, Dayananda Sagar College of Engineering, Bengaluru, 560111 India; 3 Department of Civil Engineering, Manipal Institute of Technology Bengaluru, Manipal Academy of Higher Education, Manipal, 576 104 Karnataka India

Retraction of: *Scientific Reports* 10.1038/s41598-025-89606-9, published online 08 March 2025

The Editors have retracted this Article due to a substantial overlap with a previously-submitted and later withdrawn manuscript that had only two authors in common. The Authors were unable to provide a comprehensive explanation for such a substantial change in authorship, in particular given the stated contributions to this work. The Editors have therefore lost confidence in the integrity of this Article.

Adithya Tantri disagrees with this retraction, and Sathvik Sharath Chandra, Rakesh Kumar, Archudha Arjunasamy, Sakshi Galagali, and Sujay Raghavendra Naganna did not respond to correspondence from the Editors about this retraction.

